# The Evolving Landscape of Continuous Glucose Monitoring Metrics in Type 1 Diabetes: Narrative Literature Review

**DOI:** 10.2196/92455

**Published:** 2026-07-23

**Authors:** Eric Williams Jr, Casey Rand, Alessandra Ayers, Cindy Ho, Ashley Dunova, David Klonoff, Juan Espinoza

**Affiliations:** 1 Stanley Manne Children’s Research Institute, Lurie Children's Hospital, 225 East Chicago Avenue, Chicago, IL, 60611, United States, 1 3122276090; 2 Diabetes Technology Society, Burlingame, CA, United States; 3 Diabetes Research Institute, Mills-Peninsula, San Mateo, CA, United States

**Keywords:** continuous glucose monitoring, CGM, time-series glucose data, glycemic variability, hemoglobin A1c, diabetes mellitus, diabetes research, narrative review

## Abstract

**Background:**

Continuous glucose monitoring (CGM) has transformed diabetes management and research by providing high-frequency data that address many of the limitations of hemoglobin A_1c_, enabling more precise clinical treatment targets and responsive trial endpoints. The richness and complexity of high-resolution time-series CGM data have spurred the development of numerous metrics for both clinical care and research applications. Beyond established metrics, there is a growing set of clinical, composite, and research-oriented measures that may support clinical decision support, intervention planning, risk stratification, and discovery-oriented research. This proliferation has created significant challenges in metric selection, interpretation, calculation, and standardization, particularly when metrics are applied across different devices, populations, software packages, and study designs.

**Objective:**

The objective is to map the current landscape of CGM metrics and address ongoing challenges in metric selection, clinical and research interpretation, and standardization. We further sought to distinguish between metrics primarily suited for routine clinical interpretation and those designed to explore more granular or multidimensional features of glycemia in research settings.

**Methods:**

We identified the literature focusing on the calculation, application, and interpretation of the following categories of CGM metrics: (1) standardized, (2) clinical, (3) emerging, and (4) composite. CGM metrics included in this study were identified from the 27 metrics included in the Diabetes Research Hub platform, additional published standardized and composite metrics, metrics used in established CGM analysis software, and emerging metrics identified during review. We narratively reviewed each metric’s definitions, calculation methods, interpretation, clinical and research utility, and strengths and limitations. In total, 102 articles were reviewed, supporting the synthesis of 36 distinct CGM-derived metrics.

**Results:**

The review identifies a fundamental divide in the CGM metric landscape. Standardized and clinical metrics, including time in range, mean glucose, coefficient of variation, and similar, prioritize simplicity and actionability. These metrics facilitate rapid decision-making in clinical settings but potentially mask granular glycemic fluctuations, event patterns, and discordance between average glucose values and variability. Emerging and composite metrics offer deeper insights into glycemic patterns, risk, and variability. However, many rely on specialized software or complex formulas, lack standardized thresholds or clear relationships to clinical outcomes, and do not have consensus methods of calculation and interpretation, limiting their adoption and hindering cross-study comparison.

**Conclusions:**

While consensus exists for core clinical metrics, the lack of standardization for complex metrics hinders research replicability and clinical translation. Bridging this gap requires moving toward consensus metric definitions, open-science frameworks, and standardized code libraries. Metric selection should be guided by intended use. Clinical metrics should be well-established, interpretable, and actionable. Research metrics should be clearly described, reproducible, and linked to meaningful outcomes. This review provides a comprehensive resource for navigating the diverse spectrum of CGM metrics, clarifying their applications and limitations to support both research and clinical investigation.

## Introduction

Diabetes mellitus affects over 500 million people worldwide [1]. Maintaining optimal glycemic control remains critical for preventing the devastating complications that characterize this disease. For decades, hemoglobin A_1c_ (HbA_1c_) has served as the gold standard biomarker for assessing long-term glycemic control, with extensive evidence linking elevated HbA_1c_ levels to increased risk of diabetes-related complications [2]. However, HbA_1c_ is a highly summarized, aggregate measure. It reflects a 2‐3 month average and lacks information about the daily glycemic excursions that patients experience [2]. HbA_1c_ cannot capture the frequency or severity of hypoglycemic episodes, postprandial excursions, or glycemic variability, all factors that impact patient safety, quality of life, and clinical decision-making.

Continuous glucose monitoring (CGM) has emerged as a transformative technology that overcomes these fundamental limitations [3-6]. CGM provides real-time, high-frequency glucose measurements through subcutaneously inserted sensors, offering an unprecedented, high-resolution view of an individual’s glycemic profile. Unlike single-point measurements, CGM systems can capture hundreds to thousands of measures daily, providing a more complete glycemic picture and revealing previously invisible patterns and fluctuations. This comprehensive view of glucose dynamics has enabled more personalized diabetes management and has fundamentally changed clinical care paradigms. Usage of CGM metrics in clinical practice has led to improved glycemic control, reduced hypoglycemia, and enhanced quality of life for people with diabetes [5,7,8]. Recently, evidence has emerged supporting the use of CGM metrics for monitoring in early-stage type 1 diabetes, and to predict disease progression and severity [9,10]. Consequently, CGM has rapidly become a standard component of diabetes care across type 1 and type 2 diabetes [11]. Beyond its clinical utility, CGM has transformed diabetes research by providing investigators with a powerful tool to characterize the glycemic effects of lifestyle and behavioral factors, identify predictors of glucose variability, and rigorously evaluate emerging therapeutic interventions [12-15].

Despite the potential of using CGM data for both clinical care and research, the volume and complexity of data generated by CGM technology present substantial analytical challenges. Thousands of time-series CGM readings per patient must be transformed into standardized, interpretable, and clinically meaningful summary metrics. To meet this challenge, numerous novel CGM-based metrics have been developed, including time-based metrics (eg, time in range [TIR]), glycemic summary statistics (eg, mean glucose and coefficient of variation [CV]), and measures to characterize excursions (eg, hyperglycemic episodes), among others. Existing CGM metrics include both well-established and widely accepted summary measures, often prioritizing simplicity and interpretability, and used to guide clinical management, as well as an extensive suite of emerging and research metrics designed to provide further insight. While consensus expert recommendations identifying core CGM metrics for clinical practice and research have been published [16-19], the quickly evolving landscape of metric development and usage in contemporary clinical research remains incompletely characterized. Specifically, there is a limited consolidated understanding of which metrics are being prioritized in clinical care and research, how these metrics are defined and calculated across different studies, and what specific outcomes they are intended to measure. These gaps lead to challenges in the interpretation and replication of CGM literature, limiting progress toward establishing standardized analytical frameworks that could accelerate both research innovation and clinical implementation [20].

Therefore, the purpose of this review is to examine recent literature to provide a comprehensive assessment of the current state of CGM metrics in diabetes care and research. Specifically, this review aims to (1) identify the full spectrum of CGM-derived metrics commonly being used; (2) document the calculation methods, threshold definitions, and applications of these metrics; (3) summarize their reported strengths, limitations, and interpretations; (4) offer clarity for researchers designing CGM protocols and analytic plans; (5) offer clarity for clinicians interpreting CGM-based evidence; and (6) facilitate the translation of complex CGM data into actionable clinical insights that improve diabetes knowledge, care, and patient outcomes.

## Methods

### Study Design

This study was conducted as a narrative review to map the evolving landscape of CGM metrics. A narrative review was selected because the main objective of this study is to identify existing CGM metrics, document their calculation methods, usability, strengths, and limitations to work toward standardization.

### Literature Search

A search of PubMed (search date: November 14, 2025) and Google Scholar was conducted to identify relevant literature. To facilitate reproducibility, the search included 2 strategies. First, we searched for the phrase, continuous glucose monitoring metrics. Next, we paired each specific CGM metric with that same phrase (eg, “[metric name] continuous glucose monitoring metrics”); the following CGM metrics were included in the search: (1) the names of each of 27 CGM metrics included as part of the Diabetes Research Hub (DRH) [21] (Tables 1 and 2), a leading open-science repository for CGM data sharing and research; (2) additional metrics identified in previously published CGM review articles [22-27]; (3) metrics calculated by well-established CGM analysis software packages [25-28]; and (4) through input from diabetes clinicians and researchers involved in this project.

**Table 1.** Standardized and clinical metrics.

Metric name	Description	Range or formula	Clinical utility
TIR^a^ [16,18,29-36]	Percentage of time glucose is 70‐180 mg/dL	% of readings and time 70‐180 mg/dL (3.9‐10 mmol/L)	Quantifies time spent within standard glycemic range.
TITR^g^ [16,18,37-39]	Stricter version of TIR, using 70‐140 mg/dL	% of readings and time 70‐140 mg/dL (3.9‐7.8 mmol/L)	Used to assess tighter glycemic control in specific populations.
TAR H^h^ [18,40]	Time spent in mild hyperglycemia	% of readings and time 181‐250 mg/dL (10.1‐13.9 mmol/L)	Measures time spent above target range but below critical threshold.
TAR VH^i^ [18,40]	Time spent in severe hyperglycemia	% of reading and time >250 mg/dL (13.9 mmol/L)	Identifies periods of very elevated glucose levels.
TBR L^j^ [18,40]	Time spent in mild hypoglycemia	% of readings and time 54‐69 mg/dL (3.0‐3.8 mmol/L)	Categorizes low glucose events that are not immediately severe.
TBR VL^k^ [18,40]	Time spent in severe hypoglycemia	% of readings and time <54 mg/dL (3.0 mmol/L)	Indicates severe hypoglycemic episodes requiring intervention.
Mean glucose [29,41]	Average glucose over time	$\frac{\sum \text{Glucose measurements}}{\text{Number of measurements}}$	Estimates overall glycemic control from CGM^b^ data.
Glucose variability (%CV) [4,18,36,37]	Degree of glucose fluctuation	$\frac{\text{Standard Deviation}}{\text{Mean Glucose}}\times 100$	Measures fluctuations in glucose levels over time.
GMI^c^ [30,31]	Estimate HbA_1c_^d^ from CGM data	GMI (%)=3.31+0.02392 [mean glucose in mg/dL] GMI (mmol/mol)=12.71+4.70587 [mean glucose in mmol/L]	Converts mean glucose to estimated HbA_1c_ over the life of the CGM sensor.
AUC^e^ [22,42,43]	Indicator of glycemic excursions in a specified period	$AUC=\sum_{i=1}^{n-1}\left(\frac{G_{i}+G_{i+1}}{2}\right)(t_{i+1}-t_{i})$	Helps compare interventions by assessing glycemic excursion and risk over time.
Percent wear time [18,44]	% of time CGM is worn	$\left(\frac{\text{Total Time CGM Active}}{\text{Total Time Period}}\right)\times 100$	Indicates completeness of CGM data collection.
Days of wear [18,44]	Number of days CGM was worn	$\frac{\%\;\text{Active Wear Time}\times \text{Total Monitoring Period (days)}}{100}$	Reflects duration of CGM usage for data reliability.
eA1c [31]	Tracking of average glycemia and the estimation of HbA_1c_	eA1c(t)=0.9512 * eA1c (t-1 d)+0.0488 * f (SMBG)	Fills the gap between infrequent laboratory HbA_1c_ tests. Highly predictive of the Hemoglobin Glycation Index (HGI).
Hypoglycemic episodes [17,45-48]	Event with glucose <70 mg/dL for ≥15 min	$N_{\text{Hypo}}=\sum_{i=1}^{n}I(G_{i}§lt;70)$	Defined by glucose thresholds and duration criteria.
Euglycemic episodes [35,49-51]	Glucose within normal range	$N_{\text{Eugly}}=\sum_{i=1}^{n}I(70\leq G_{i}\leq 180)$	Identifies periods of normoglycemia. May include atypical conditions like EDKA^f^
Hyperglycemic episodes [18,52-55]	Glucose >180 mg/dL for ≥15 min	$N_{\text{Hyper}}=\sum_{i=1}^{n}I(G_{i}§gt;180)$	Categorizes elevated glucose events based on severity.

a TIR: time in range.

b CGM: continuous glucose monitoring.

c GMI: glucose management indicator.

d HbA_1c_: hemoglobin A_1c_.

e AUC: area under the curve.

f EDKA: euglycemic diabetic ketoacidosis.

g TITR: time in tight range

h TAR H: time above range high

i TAR VH: time above range very high

j TBR L: time below range low

k TBR VL: time below range very low

**Table 2.** Emerging and composite metrics.

Metric name	Description	Range or formula	Clinical utility
MAGE^a^ [36]	The “gold standard” for assessing short-term within-day glycemic variability.	MAGE = $\sum \frac{\lambda }{\chi }\;\text{if }\lambda §gt;v$ λ=blood glucose changes from peak to nadir =number of valid observations V=1 SD mean glucose for 24-h period	It is used as a measure of glycemic lability, though the LI^b^ may correlate more closely with clinical assessment of lability.
CONGA^c^ [37,38,56]	A novel method developed to assess intraday glycemic variability.	CONGA=$\sqrt{\frac{\sum_{t=-t_{1}}^{t_{1}}(D_{t}-\bar{D}{)}^{2}}{k-1}}$ $D=\frac{\sum_{t=t_{1}}^{t_{k}}D_{t}}{K}\;D_{t}=G_{t}-G_{t-m}$ k=number of observations with an observation n×60 min ago m=n×60 G=glucose measured	Distinguishes between patterns of glycemic control.
Mean of daily differences [12,37,42,57]	Measure of day-to-day variability.	k=number of observations with an observation 24 h ago G=glucose measured t=time (in min)	Determines if the short-term CGM^d^ trace is representative of the 3-month glycemic pattern.
LI [4,41]	A measure of glycemic lability.	$LI=\sum_{n=1}^{N-1}\frac{(G_{t}-G_{n+1}{)}^{2}}{\left(t_{n+1}-t_{n}\right)}$ k=number of observations with an observation 24 h ago n=total number of readings in a week t=time	Used to quantify labile glucose control. It correlates more closely than MAGE with the clinical assessment of lability.
GCI^e^ [42]	A novel measure developed to quantify the shape of glycemic variability.	Calculate and extract log-periodogram summary. Calculate the GCI by deriving the weights from the summary. Apply canonical correlation analysis to the weights to produce a raw GCI score. $GCI\propto \sum_{i=1}^{6}w_{i}m_{i}$	Used for the analysis of the shape of glycemic variability.
GRI^f^ [43,58]	A single-number summary that assesses the quality of glycemia from CGM tracings.	Hypoglycemia component =VLow+(0.8×Low) Hyperglycemia Component =VHigh+(0.5×High) GRI = (3.0×VLow) + (2.4×Low) + (1.6×VHigh) + (0.8×High)	Assesses glycemic quality in clinical practice. Its primary benefit is to quickly evaluate glycemic control.
M value [34,37]	An index used to describe glucose variability and the quality of glycemic control.	$M=\frac{\sum_{t=t_{1}}^{t_{k}}{\left|10\times \log\frac{G_{t}\times 18}{IGV}\right|}^{3}}{N}$ G=glucose measured IGV=ideal glucose values K=total number of observations N=total number of readings	Risk marker used to assess the quality of glycemic control.
J Index [24,46,59,60]	A universal index proposed for the assessment of current glucose control in diabetic patients.	J=0.001(MBG + SD))^2^ for glucose measured in mg/dL J=0.324(MBG + SD)^2^ for glucose measured in mmol/L	Highly sensitive to both the glycemic level and glycemic variations.
GRADE^g^ [22,37,61]	An integrated risk score that summarizes the clinical risks of both hypoglycemia and hyperglycemia.	mmol/L GRADE value =$425\times {\left[\log(\log(X))+0.16\right]}^{2}$ mg/dL GRADE value =$425\times {\left[\log(\log(X\times 18))+0.16\right]}^{2}$ Where X=blood glucose	Used as an adjunct to HbA_1c_^h^ to report the degree of risk associated with glycemic variability.
ADRR^i^ [33,62]	A measure used to assess blood glucose variability.	ADRR =$\frac{1}{N}\sum_{t=1}^{N}[LR+HR]$ N=total number of readings LR=risk value attributed to low glucose HR=risk value attributed to high glucose	Adequate indicator of hyperglycemia.
LBGI^j^ [29]	Quantifies the frequency and extent of low blood glucose readings to provide an optimal prediction of hypoglycemia risk.	LBGI =$\frac{1}{n}\sum_{i=1}^{n}rl(\chi_{i})$	Predicts the risk of severe hypoglycemia and helps split overall glucose variation into hypo- and hyperglycemia risk components.
HBGI^k^ [4,52,56,57]	Quantifies the frequency and extent of high blood glucose readings to predict the risk of hyperglycemia.	HBGI =$\frac{1}{n}\sum_{i=1}^{n}rl(\chi_{i})$	Predicts the risk of hyperglycemia, correlates with HbA_1c_, and is associated with hyperglycemic excursions.
IGC^l^ [23,24]	Composite measure of overall glycemic control.	IGC =Hypoglycemia Index + Hyperglycemia Index	Provides a comprehensive metric that is highly sensitive for detecting improvements in glycemic control following therapeutic interventions.
HAS^m^ [22,59]	Combines hypoglycemia incidence and HbA_1c_ change.	Change in A_1c_, the rate of SH^n^ events, and a change in the rate of SH events.A score of HAS≥75 is arbitrarily assigned to represent a good to excellent intervention.	Allows for easier comparison of qualitatively different diabetes interventions using a single score.
Qscore [22,60]	A composite metric used to evaluate CGM profiles and screen for profiles needing therapeutic action.	Q-Score =$\begin{array}{rl} 8 & +\frac{MBG-7.8}{1.7}+\frac{range-7.5}{2.9}+\frac{tG§lt;3.9-0.6}{1.2}\\ +\frac{tG§lt;8.9-6.2}{5.7}+\frac{MODD-1.8}{0.9} \end{array}$ Very Good:<4.0; Good: 4.0‐5.9; Satisfactory: 6.0‐8.4; Fair: 8.5‐11.9; Poor:≥12.0	Stratifies metabolic control (eg,<4.0 is “very good”; ≥12.0 is “poor”); identifies individual CGM parameters that require optimization for patient-tailored therapy.
PGS^o^ [22]	Combines TIR^p^, mean glucose, Glycemic Variability Percentage (GVP), and the frequency and severity of hypoglycemia episodes.	PGS =f(GVP)+ g(MG)+ h(PTIR)+ j(N54,N70)	Identify problem areas in glycemic control to guide modification of therapy and complements A_1C_ assessment.
The Hypo-Triad [22,61]	Integrates hypoglycemic AUC^q^, duration/time, and frequency of episodes to quantify the severity and risk of hypoglycemia.	IntHypo=|AUCxTime|=$\sqrt{AUC^{2}+Time^{2}}$	IntHypo describes the immediate clinical impact, while HypoRV represents the patient’s overall hypoglycemic environment, providing supplementary insights.
Glucose Pentagon and CGP^r^ [22,62]	Provides a visual pentagonal shape and numerical score by integrating five glucose-centric CGM variables.	The area (ACGP) is calculated by summing the areas of the five resulting 72° triangles: ACGP =AToR CV+ ACV-IntHYPO+ AIntHYPO-IntHYPER+ AIntHYPER Mean glucose + AMean glucose-ToR, where:Ax-y = ½ a×b×sin γ (*γ*=angle between the axes, each 72°;a, b=length of each axis; A=area of triangle; and,x-y=length of axis for each area).	Enables visual and numerical comparison of glycemic control against an idealized non-diabetic state.
COGI^s^ [22]	Evaluates the quality of control using TIR, TBR^t^, and SD.	Index ranges between 0 and 100TIR (50% weight), TBR <70 mg/dL (35% weight), and SD (15% weight). %CV^u^ may be used instead of SD	Provides a focused assessment on the 3 components considered most crucial for achieving safe glycemic control.
Acute Glycemic Gap (GMI-HbA_1c_) [46]	An independent risk factor for longer inpatient stays.	GMI-HbA_1c_	Captures the discrepancy between patients' CGM readings and their HbA_1C_.

a MAGE: mean average glucose excursions.

b LI: lability index.

c CONGA: continuous overall net glycemic action.

d CGM: continuous glucose monitoring.

e GCI: glucose color index.

f GRI: glycemic risk index.

g GRADE: glycemic risk assessment diabetes equation.

h HbA_1c_: hemoglobin A_1c_.

i ADRR: average daily risk range.

j LGBI: low blood glucose index.

k HGBI: high blood glucose index.

l IGC: index of glycemic control.

m HAS: hypoglycemia score.

n SH: severe hypoglycemia.

o PGS: personal glycemic state.

p TIR: time in range.

q AUC: area under the curve.

r CGP: comprehensive glucose profile.

s COGI: continuous glucose monitoring index.

t TBR: time below range.

u %CV: glucose variability.

### Article Screening

A standardized screening process was developed to assess the relevance of retrieved articles. Screening information for each article, including the article objective, CGM-derived metrics discussed, and details regarding metric calculation, handling, and relevance, was recorded in Airtable (Formagrid Inc), a cloud-based relational database platform used to organize, link, and track project records. Moreover, 3 team members reviewed the titles, abstracts, and criteria questions to determine each article’s relevance to the aim of the study. Discrepancies in inclusion were resolved through consensus or consultation with an additional lead researcher (refer to Authors’ Contributions section). Articles were included for full-text review if they reported on the use, handling, or calculation of CGM-derived metrics. The results of the data extraction process are detailed in the results section.

## Results

### Literature Search and Article Screening

The search and screening process resulted in 102 articles flagged for full review, with article dates spanning from 1995 to 2025. Table 3 describes the standardized fields captured in the data extraction process. The included articles provided the foundational data for key details highlighting the calculation, relevance, definitions, and functionality of 36 CGM metrics. Data on each metric were summarized across articles by expert review.

**Table 3.** Data extraction fields captured for all included articles.

Focus and data field	Description
Article	
Study objective	The main research objective or primary endpoint of the study.
Prioritized metrics	The CGM^a^ metrics highlighted as primary outcomes or key findings.
Relevance	A summary of the article’s direct relevance to the review’s objectives.
Technology	The CGM devices and software tools used for data collection and analysis.
Metric	
Definition	Purpose and detailed definition of the metric.
Range and interpretation	Published ranges of the metric and the interpretation of these values.
Formula	The specific formula or methodology used to calculate the metric.
Author recommendations	Author recommendations regarding the use, interpretation, strengths, and limitations of the metric.

a CGM: continuous glucose monitoring.

### Overview of Identified CGM Metrics

Our review of the literature identified a total of 36 distinct CGM-derived metrics currently used for either diabetes clinical care, research, or both. Based on their application, calculation complexity, and degree of adoption, these metrics were categorized into two primary groups: (1) standard and clinical metrics and (2) emerging and composite metrics.

### Standardized and Clinical Metrics

#### Overview

The standardized and clinical metrics (Table 1) comprise a core set of measures that have achieved widespread consensus and adoption. These metrics form the foundation of modern diabetes management, prioritizing simplicity, actionability, and interpretability for real-world diabetes management. They are designed to be easily understood by both health care professionals and people with diabetes, optimized for rapid interpretation during patient visits to enable real-time clinical decision-making. Overall, these metrics align well with established diabetes management practices and can be readily incorporated into existing clinical workflows and electronic health records. Standardized and clinical CGM metrics can be broadly categorized into several distinct categories described below, each serving specific purposes in diabetes care.

#### Composite Visual Reports

The ambulatory glucose profile (AGP) provides a standardized, single-page visual report of CGM data [18,22], summarizing key metrics, including composite indices like the glucose management indicator (GMI), facilitating the clinical interpretation of overall glycemic quality [17,58]. Daily glucose profiles are now typically presented in the AGP for several days [12,63] to aid in identifying typical 24-hour glucose patterns across treatment periods [18,63].

#### Time-Based Glycemic Metrics

These metrics quantify the percentage of time spent within defined glucose thresholds [18], and are widely used in clinical care [18,45]. Key metrics include TIR, time below range TBR; low <70 mg/dL and very low <54 mg/dL, and time above range TAR; high >180 mg/dL and very high >250 mg/dL [18]. A primary limitation is the inherent bias toward hyperglycemia due to the asymmetrical distribution of blood glucose values, which gives disproportionately heavier weight to high excursions in statistical calculations [29]. Consequently, metrics impacted by both low and high measures of glycemia, such as TIR, may lack sensitivity to changes in low values, such as longitudinal improvements in TBR. This limitation highlights the inadequacy of time-based measures in capturing the full clinical risk associated with hypo- or hyperglycemia as compared with composite indices [58].

#### Summary Statistical Metrics

These metrics characterize overall and long-term glycemic exposure, reflecting average glucose and stability over periods typically ranging from weeks to months [23]. These measurements include mean glucose, glucose variability (commonly %CV), and the estimated average measure GMI. This category also includes critical data sufficiency metrics such as percent wear time and days of wear. A significant limitation of summary metrics, like mean glucose and GMI, is that they do not reflect glucose fluctuations or glycemic variability [22,29,45,63], meaning significantly varied glucose profiles can yield identical mean values [63]. GMI often shows clinically significant discordance with measured HbA_1c_ (≥0.5%, 6 mmol/mol) due to nonglycemic factors influencing red blood cell turnover, reinforcing caution against interpreting GMI as interchangeable with HbA_1c_ [18,30,31]. Studies introduced an updated calculation of the GMI, known as the updated glucose management indicator (uGMI). The uGMI is derived from a kinetic model, and it is the expected HbA_1c_ under a steady glucose level [32]. Furthermore, estimates of glycemic variability often have a larger relative error compared with estimates of mean glucose [23] due to their sensitivity to noise and extreme values.

#### Event-Based Metrics

These metrics focus on discrete episodes of acute glycemic extremes, providing actionable data crucial for assessing patient safety and guiding immediate therapeutic adjustments [17,18,33,63]. Unlike time-based metrics, these metrics include quantifying dangerous excursions such as hypoglycemic events (levels 1 and 2, eg, <70 mg/dL and <54 mg/dL) and hyperglycemic events (levels 1 and 2, eg, >180 mg/dL and >250 mg/dL) [18], alongside critical metabolic incidents (eg, euglycemic diabetic ketoacidosis). A primary limitation of event-based metrics is that CGM accuracy is often lower in the hypoglycemic range [17,18,34]. Also, the precise calculation of the number of events requires strict adherence to standardized definitions regarding the specific glucose thresholds and the required minimum duration (≥15 min) that must be maintained below or above the threshold [45], and subsequent events must be separated by a return to the target range for a specified time (eg, ≥15 min above 70 mg/dL) [17]. Without these standards, definitions can lead to over- or underestimation of event incidence [17]. For diagnosing acute events during periods of apparent euglycemia, such as euglycemic diabetic ketoacidosis, relying solely on glucose monitoring is insufficient, necessitating separate monitoring of metabolic acidosis and ketones [35]. Evaluating the frequency of hypoglycemic and hyperglycemic events is often considered more clinically meaningful than solely reporting the percentage of time spent in the respective ranges [45].

### Emerging and Composite Metrics in Research Settings

While standard clinical CGM metrics are widely used in both research and clinical practice, emerging and composite metrics are gaining interest due to their potential to capture more complex aspects of glycemic control. Given the complexity of these metrics, variability in definitions, and evolving clinical relevance, it is challenging to compare results across different studies. These metrics often require specialized knowledge or software for calculation and interpretation, which limits their practical application in day-to-day clinical care. Furthermore, they may not have clear, immediate implications for treatment adjustments in routine clinical care. Metrics such as glycemic risk index (GRI) and glycemic risk assessment diabetes equation (GRADE) may eventually transition to clinical use as validation grows, but currently remain secondary to broadly endorsed measures. This review organizes the emerging and composite metrics in three categories: (1) metrics of glycemic variability, (2) composite indices of glycemic quality and risk, and (3) discordance metrics. This section provides a more detailed overview to aid interpretation and highlight their potential utility in future research and clinical decision-making. Table 2 summarizes each emerging and composite metric included in this review.

### Metrics of Glycemic Variability

#### Mean Amplitude of Glycemic Excursions

Mean average glucose excursions (MAGE) focuses on the minimum to maximum span of blood glucose levels and is widely considered the gold standard for assessing short-term glycemic variability [36]. It was developed as a measure to characterize glycemic variability specifically for postprandial glucose excursions above the mean glucose level. MAGE calculates the mean of glycemic excursions by identifying nadir to peak blood glucose levels. The amplitude from peak to nadir glucose averages that exceed 1 SD of glucose are included in the final MAGE calculation.

Previously, researchers evaluated MAGE at multiple 2-day periods within a 14-day CGM sensor lifespan, finding these equivalent to MAGE calculated from longer (3‐4 day) periods [36]. The study suggested that short-term periods are sufficient. MAGE lacks standardization in its calculation. There is no standard defining whether the amplitude should be calculated using ascending limbs, descending limbs, or a weighted average of both limbs. It requires specialized software programs for accurate calculations, limiting the metric’s ability to be used in real-time practice or integrated into electronic health records.

#### Continuous Overall Net Glycemic Action

The continuous overall net glycemic action (CONGA) was originally developed for data from continuous interstitial glucose monitoring [37,38], and can be described as an index of the amount of time spent in glycemic excursions and the degree of glycemic variation that an individual experiences [37]. CONGA is particularly useful for helping clinicians and patients understand the magnitude and timing of blood glucose fluctuations in order to understand and optimize the patient’s degree of glycemic control. The normative reference range for CONGA (mean +/− 2 SD) for healthy patients is 3.6‐5.5, but may vary slightly by different ethnicities [39]. CONGA includes all data points, which means it may fail to effectively separate meaningful glycemic excursions from data noise [37].

#### Mean of Daily Differences

The mean of daily differences (MODD) was originally developed as a measure of intraday glycemic variability [12]. MODD is calculated using pairs of blood glucose measurements from CGM that are 24 hours apart. MODD was originally introduced as a metric to be used to better understand the magnitude of glycemic variability through CGM data. MODD is highly correlated with other measures of variability, such as CONGA and SD. The average normative reference range for MODD for healthy patients is 0.0‐3.5 (SD 2), varying slightly for different ethnicities [39].

#### Lability Index

The lability index (LI) is designed to quantify glycemic variability by assessing fluctuation in glucose levels over time. It is particularly useful for identifying periods of significant glucose variability, which can be critical for managing diabetes effectively. The LI formula assesses variability in 3 consecutive glucose values at a time [4]. High LI indicates greater variability and potential instability in glucose levels, which can be associated with increased risks of hypoglycemia and hyperglycemia. Researchers compared the outcome of the HYPO score and the LI in patients living with type I diabetes who were undergoing islet transplant therapy [41]. The HYPO score is a measurement based on the frequency, severity, and degree of unawareness of a hypoglycemic event. Posttransplant patients experienced a significant reduction in the HYPO score and LI, indicating clinical relevance of LI as an objective measure for assessing the severity of glycemic lability [41]. The LI also had a high correlation with the metric, MAGE. This correlation makes it challenging to statistically isolate the unique and independent contribution of liability to long-term risk assessment [58]. The LI also requires specialized software to address complex calculation methods.

#### Glucose Color Index

Standard CGM metrics are inadequate in representing the shape of glucose variability [42]. The glucose color index (GCI) is a new CGM metric that captures the shape of glucose variability. GCI uses periodogram signal decomposition. The metric was created through a multistep process involving the calculation of log-periodograms for each CGM time series, summarization using disjoint piece-wise linear models in 3 frequency regions, and refinement into a single value using canonical correlation analysis. The GCI is calculated by applying a linear combination of weights to the 6-parameter log-periodogram summaries, with these weights derived to maximize the test-retest correlation within participants over a 3-month period. Raw GCI values are then standardized to a scale of 0‐100 using a reference population for interpretability. In the study, the GCI was used to assess its reliability by comparing its 3-month test-retest correlation (*R*=.75) with standard CGM metrics and evaluate associations with diabetes comorbidities in older adults with type 2 diabetes [42]. The GCI showed significant associations (*P*<.05) with impaired physical functioning, frailty or prefrailty, cardiovascular disease, chronic kidney disease, and dementia or mild cognitive impairment after adjustment for confounders [42]. Overall, the study explains how the GCI captures clinically relevant information about glucose dysregulation that is not fully captured by standard CGM metrics. The GCI metric creates space for the development of CGM metrics that more fully incorporate time series information.

Overall, while metrics of glycemic variability provide granular insights into glucose stability and fluctuations that standard averages miss, their application is currently hindered by a lack of computational standardization and the frequent need for specialized software.

### Composite Indices of Glycemic Quality and Risk

#### Glycemic Risk Index

The glycemic risk index (GRI) aims to assist with basic clinical interpretation of CGM data. To develop the GRI, researchers compiled a dataset of 14-day CGM tracings from 225 insulin-treated adults with diabetes [58]. Using a balanced incomplete block design, 330 clinicians with extensive experience in CGM analysis and interpretation ranked these tracings based on the quality of glycemia [58]. Principal component analysis and multiple regressions were used to create a model predicting clinician rankings using the following 7 standard metrics from an AGP: very low-glucose and low-glucose hypoglycemia, very high-glucose and high-glucose hyperglycemia, TIR, mean glucose, and CV. The resulting GRI provides a single-number summary of glycemic quality, with its hypoglycemia and hyperglycemia components offering actionable scores. Additionally, the GRI grid, a graphical display, enables clinicians and researchers to visualize and determine the glycemic effects of prescribed and investigational treatments, enhancing its utility in both clinical practice and research settings [43]. The GRI is based on clinician rankings rather than clinical outcomes. Additional studies are necessary to determine how accurately GRI predicts long-term complications. Furthermore, the GRI does not inherently distinguish glycemic risks by time of day (eg, daytime vs nighttime), which is crucial for making precise therapeutic adjustments [58].

#### M-Value

The M-value provides valuable insights into glycemic control and variability. It is defined as the difference between the observed blood sugar and normal blood sugar [37]. Lower M-values indicate better glycemic control, as demonstrated in a study comparing insulin glargine and insulin degludec in patients with type 1 diabetes using CGMs [44]. The researchers found that M-values were significantly smaller in patients treated with insulin degludec compared with those treated with insulin glargine, suggesting improved glycemic variability with insulin degludec. This study highlights the utility of the M-value in assessing the effectiveness of different insulin treatments and its role in evaluating glycemic control in clinical research [34]. A fundamental limitation is that the M-value is a hybrid measure that reflects both glucose variability and mean glycemia. It does not isolate variability itself [37]. Additionally, calculating the M-value requires complex calculation programs, which limits immediate application in acute settings like the ICU [46].

#### J-Index

The J-index was proposed as an improvement over the existing M-value, integrating both the mean blood glucose (MBG) and SD of blood glucose to provide a comprehensive representation of glycemic status [59]. By incorporating these 2 components, the J-index effectively captures both the mean level and variability of glycemia, addressing key aspects of glucose control. Moreover, 1 study applied the J-index to evaluate glycemic control in a group of patients with type 1 diabetes undergoing intensive insulin therapy [59]. Researchers used the J-index to assess the effectiveness of different insulin regimens and found it to be a valuable tool for comparing glycemic control between patient groups and monitoring individual progress over time [24,46,60]. Similar studies demonstrated the practical application of the J-index in clinical settings and its potential to provide meaningful insights into glucose management strategies for diabetic patients. Similar to the M-value, the J-index requires the use of specialized software or programming, thereby hindering its immediate application in clinical care settings.

#### GRADE

The GRADE score is an innovative metric designed to comprehensively evaluate glycemic control in patients with diabetes [22]. GRADE combines 2 risk quantities to summarize the overall risk associated with both hypoglycemia and hyperglycemia. The score’s calculation involves a log-log transformation, with risk increasing as glucose levels deviate from an assigned nadir of 90 mg/dL. This approach provides a clinically meaningful measure of glycemic risk, applying different weights to hyperglycemic and hypoglycemic values.

The development of GRADE stemmed from the increasing practice of multiple daily glucose assessments in patients with diabetes and the need for a more comprehensive evaluation of glycemic control [61]. To create this methodology, Hill et al [50] collaborated with 50 diabetes professionals who assigned risk values to a range of 40 blood glucose concentrations. From these responses, a generic function of glycemic risk was devised and applied to patient glucose profiles to generate an integrated risk score.

Interpreting GRADE scores is straightforward, with values below 5 corresponding to euglycemia. The simplicity of generating GRADE scores from any blood glucose profile makes it a valuable tool for clinicians. It can be used in conjunction with HbA_1c_ to provide a more comprehensive assessment of glycemic control, offering insights into the degree of risk associated with glycemic variability that HbA_1c_ alone cannot capture. The GRADE score does not directly measure glucose fluctuations. The derivation results in an expression that often reflects quasi-mean glycemia or a frequency distribution [37]. A high score can be generated by either hyperglycemia or hypoglycemia. Therefore, the formula does not explicitly specify the nature of the associated risk [37].

#### Average Daily Risk Range

The average daily risk range (ADRR) was created to serve as a single metric to summarize blood glucose variability [51]. The ADRR is a diabetes-specific measure designed to assess the risk of both hyperglycemia and hypoglycemia. It is calculated using mathematically transformed glucose levels to give equal weight to high and low blood glucose excursions [52]. ADRR requires at least 14 days of blood glucose data with a minimum of 3 checks per day, although some studies have used fewer days. The metric is scored based on risk categories: low risk (0‐19), moderate risk (20-40), and high risk (40 and above) [62].

A study used ADRR to assess glycemic variability in children aged 2‐6 years with type 1 diabetes [33]. The researchers found that 72% of the children’s ADRR values fell into the “high-risk” range when using adult guidelines, suggesting a need for pediatric-specific ADRR standards [33]. The study also revealed that ADRR was highly correlated with indicators of hyperglycemia but only weakly correlated with measures of hypoglycemia in this young population [33]. Some articles in this review highlighted ADRR’s broader applications in clinical settings [62]. It emphasized ADRR’s ability to provide meaningful data on patients’ risk for both hyperglycemia and hypoglycemia, offering insights not available from HbA_1c_ values alone. The review also noted that research has shown ADRR to be a reliable predictor of extreme blood glucose values across different diabetes types and patient ages.

#### Low Blood Glucose Index

Low blood glucose index (LBGI) was developed specifically to measure the risk associated with low blood glucose events. There are four distinct risk categories of the LBGI: (1) minimal risk for hypoglycemia (LBGI≤1.1), (2) low risk (1.1<LBGI≤2.5), (3) moderate risk (2.5<LBGI≤5), and (4) high risk (LBGI>5) [29]. The LBGI has been demonstrated to predict severe hypoglycemia, while the high blood glucose index (HBGI) is associated with HbA_1c_ levels and predicting hyperglycemic excursions. Additionally, the ADRR serves as a measure of overall glucose variability, capturing the risk of both hypoglycemia and hyperglycemia.

LBGI's primary strength is its ability to quantify the frequency and extent of low blood glucose excursions, placing more emphasis on the hypoglycemic risk [44]. It has been shown to be a robust predictor of symptomatic hypoglycemic episodes [54]. Because LBGI correlates well with the occurrence of severe hypoglycemia, it is a valuable tool for identifying people at higher risk of hypoglycemia episodes. However, LBGI only focuses on hypoglycemia and does not provide information about overall glycemic control or hyperglycemic events. Additionally, interpreting LBGI values may require a higher level of clinical experience as well as clinical context to translate into meaningful treatment decisions.

#### High Blood Glucose Index

High blood glucose index (HBGI) was designed to assess the risk associated with hyperglycemic events. Like LBGI, the HBGI quantifies glycemic variability focusing on hypoglycemic excursions. The cutoff points for the HBGI (4.5 and 9) are used to categorize the risk levels low, moderate, and high for hyperglycemia [56]. Risk scores above 0 are defined as HBGI [4]. In a study evaluating the improvement in glucose variability and its correlation with baseline HbA_1c_ and hypoglycemic events, the HBGI was found to have the largest proportional reduction (65.5%) among several glucose variability metrics after 24 weeks of treatment intensification. Furthermore, the HBGI was identified as the metric most predictive of improvements in HbA_1c_ levels [56].

This metric can be used to quantify the frequency and extent of high blood glucose excursions and can provide insights into the risk of hyperglycemia-related complications. HBGI has been shown to positively correlate with HbA_1c_, making it useful for estimating overall glycemic control and potential long-term outcomes [57]. Similar to LBGI, a limitation of HBGI is that it provides a unidimensional view of glycemic control. Furthermore, the interpretation of HBGI values may not be intuitive for all people with diabetes and health care professionals, which may require additional education for effective use in clinical practice.

#### Index of Glycemic Control

Index of glycemic control (IGC) assesses the overall quality of glycemic control [23,24]. It is calculated as the sum of the hypoglycemia index and the hyperglycemia index, assigning more weight to severe hypo- or hyperglycemic values [22,24]. One study concluded the IGC to be one of the most sensitive criteria for detecting responses to therapeutic intervention, such as the unmasking of data [24]. A disadvantage of using IGC as a composite measure is that it relies solely on hypoglycemia and hyperglycemia components, which possibly excludes other valuable metrics [24].

#### Hypoglycemia Score

This metric is primarily based on a change in severe hypoglycemic events. Hypoglycemia score (HAS) aims to express overall glycemic control by simultaneously accounting for 2 key factors: the incidence of hypoglycemia and the change in HbA_1c_ [22]. HAS provides a method for comprehensive assessment and does not require complex data or equations. Its current framework uses subjective weightings and lacks CIs, meaning the results are arbitrary and there is no assurance of reproducibility from one observer to the next [22].

#### Q-Score

The Q-score was developed to automate objective evaluations of CGM profiles and serve as a screening tool to identify patients who require therapeutic intervention [60]. It is calculated using MBG, TBR, TAR, within-day variability, and MODD for between-day variability. Q-score values range from poor (≥12.0) to very good (<4.0) [22]. The examination of components that contribute to the Q-score allows clinicians to identify weak points in glycemic control. The Q-score is similar to a difficulty rating for a task. The score significantly increases with the complexity of treatment. A limitation of the Q-score is that there is no evidence on its use of MODD offering a better insight into glycemic variability than standard measures, such as SD or CV [60].

#### Personal Glycemic State

Personal glycemic state (PGS)is designed to provide a simple measure for quickly identifying problem areas in glycemic control [22]. It is calculated using TIR, mean glucose, glycemic variability percentage, and the number of hypoglycemic episodes at <70 mg/dL and <54 mg/dL thresholds [22]. The score is used to complement HbA_1c_ by measuring glycemic control. However, a limitation is that it does not include hyperglycemia as a component, and considers the frequency of glucose oscillations, making it partially a measure of frequency stability rather than exclusively magnitude [22].

#### The Hypotriad

The hypotriad uses 3 components within a 3D space model to assess the severity and risk associated with hypoglycemia. Its components are area under the curve (AUC), time spent in hypoglycemia, and the frequency of hypoglycemic episodes per day [62]. These core metrics are integrated to calculate two related composite metrics known as (1) the intensity of hypoglycemia (IntHypo), defined as a vector of AUC and time, and (2) the hypoglycemia risk volume, which is the product of IntHypo and the episode rate [62]. A major limitation of the hypotriad is that it focuses exclusively on hypoglycemia and does not include any components describing hyperglycemia, which is an important factor for assessing overall glycemic control [22].

#### Glucose Pentagon and Comprehensive Glucose Pentagon

The glucose pentagon and comprehensive glucose pentagon (CGP) are composite metrics designed to provide a simultaneous numerical and visual representation of glycemic control [22]. The original glucose pentagon integrated 5 axes, including MBG, metrics for hyperglycemic AUC, time in hyperglycemia, SD, and HbA_1c_ [22]. The CGP modified this by eliminating HbA_1c_ and introducing mean glucose, %CV, time out of range, the intensity of hyperglycemia (IntHyper), and the intensity of hypoglycemia (IntHypo), with %CV replacing SD to account for abnormal glucose distributions and independence from mean glucose. The core output of the CGP is the area of the resulting pentagon, which increases as glycemic control worsens relative to a person without diabetes (area normalized to 1) [22]. A key limitation is the complexity of the nonstandard metrics like IntHyper and IntHypo, which use quadratic averages of AUC and duration, making them difficult for most clinicians to visualize and interpret [22].

#### CGM Index

Continuous glucose monitoring index (COGI) evaluates the quality of glucose control in patients with type 1 diabetes [22]. It is calculated using 3 weighted components: TIR, TBR, and SD, with TIR carrying the most weight [22]. COGI provides a focused view on the 3 components, also allowing for %CV to be used in replacement of SD to measure variability. A potential limitation of COGI is that its component weighting was determined by expert opinion and it does not account for TBR very low (levels below 54 mg/dL) [22].

Overall, composite indices of glycemic quality and risk attempt to simplify multidimensional CGM data into unified risk scores for easier comparison, yet their widespread clinical adoption is limited by complex, nonstandardized definitions and a frequent reliance on expert opinion rather than hard clinical outcomes.

### Discordance Metrics: Acute Glycemic Gap

Acute glycemic gap (GMI−HbA_1c_) is the difference between the GMI and HbA_1c_. This metric was created to capture the discrepancy between a patient’s recent glucose levels captured by CGMs and their HbA_1c_. GMI−HbA_1c_ is calculated by subtracting the actual HbA_1c_ from the GMI-derived CGM data. The study found that this metric was significantly associated with clinical outcomes in patients in the ICU. Specifically, it showed a positive correlation with the duration of ICU stay (*r*=0.533, *P*<.001) and was significantly higher in nonsurvivors at 28 days compared with survivors (mean 2.0, SD 1.3 vs mean 0.0, SD 1.6; *P*<.001) [46]. After adjusting for confounding factors, GMI-HbA_1c_ remained an independent risk factor for longer ICU stay (coefficient=2.34, 95% CI 0.54‐4.14; *P*=.02) and higher 28-day mortality rate (hazard ratio 2.42, 95% CI 1.01‐5.76; *P*=.046). The authors concluded that GMI-HbA_1c_ is an independent risk factor for longer ICU stay and 28-day mortality rate [46], suggesting that CGM might be beneficial for critically ill patients in the ICU setting, regardless of whether they have diabetes.

By quantifying the discrepancy between CGM-derived estimates and laboratory biomarkers, discordance metrics can help identify independent risk factors for adverse outcomes and highlight critical nonglycemic physiological variances that impact assessment.

## Discussion

This narrative review mapped the current landscape of 36 CGM metrics, providing a comprehensive resource to navigate between CGM metric appropriateness for clinical simplicity and research complexity. The standard clinical metrics, such as TIR and GMI, have achieved widespread adoption due to their simplicity, interpretability, and direct correlation with established clinical metrics such as HbA_1c_ as well as long-term complications. These metrics can be used for daily glucose management decisions and are ideal for use in time-constrained clinical settings, offering easy-to-digest snapshots of glycemic control. However, these metrics may mask underlying glycemic variability and fail to identify more granular patterns that could improve patient outcomes.

The expanding ecosystem of emerging and composite metrics identifies and summarizes glycemic patterns using composite risk scoring (eg, GRI, GRADE, and ADRR) and granular analysis (eg, MAGE, MODD, and CONGA). These metrics involve more complex formulas that offer mechanistic insights, which allow researchers to characterize glycemic health more fully, offering insight into daily fluctuations. This increased granularity often comes with increased complexity. Developing intuitive visualization and standard reports of such metrics, such as the existing AGP, has the potential to increase the usage of these metrics in the future.

A critical finding of this review is the lack of standardization in how complex metrics are calculated. Even well-known metrics, such as MAGE, are highly sensitive to data processing decisions, such as the specific software package used. Inherent differences in CGM sensor hardware, including differences in sampling frequency (eg, 1-min vs 5-min intervals), add to the complexity of standardizing metric calculation. This variability means that scores in one study may not be comparable with another. This lack of standardization and reproducibility is a major barrier to universal metric adoption and undermines the validity of CGM research. To address this, the field must move toward open science frameworks [21] and adoption of standardized code libraries [25-28] to ensure that definitions and interpretations of metrics are consistent across devices and trials. Consensus standardization, implementation, and interpretation of such metrics will need to be established before their potential for impact can be fully realized.

This review has several limitations. First, the search strategy was restricted to articles published in English and indexed in PubMed or Google Scholar. Studies published in other languages or limited to other databases may have been missed. Second, our primary search terms included the 27 metrics identified by the DRH [21]. This prespecified list may exclude niche or newly proposed metrics from this quickly evolving landscape that have not yet achieved broader recognition. Third, the studies included were published across nearly a 25-year period. CGM hardware and software have evolved dramatically in this time. Articles included therefore used a heterogeneous mix of CGM devices ranging from early-generation sensors to contemporary systems. Evolving sensor accuracy and sampling frequency across these generations can influence the calculation and precision of the reported metrics, making assessment of metrics challenging. Furthermore, the increase of complex metrics can unknowingly widen health disparities, particularly for patients and nonspecialist clinicians. While this narrative review highlights the breadth of available CGM metrics, many of these metrics require specialized knowledge, software, and/or large amounts of data for interpretation. This requirement poses a significant barrier for individuals with low digital or health literacy. Finally, this review focused primarily on the definitions and calculations of metrics. An evaluation of the strength of evidence linking each emerging metric to hard clinical outcomes was beyond the scope of this paper.

As CGM technology continues to advance and become more accessible, the role of CGM metrics in diabetes management is likely to grow. Future research may focus on refining existing metrics, developing new ones, and further elucidating their relationships with clinical outcomes. While consensus exists for core clinical metrics, the lack of standardization for more complex metrics remains a barrier to universal translation. The value of CGM metrics lies in their ability to provide reliable day-to-day insights, playing a crucial role in breakthrough discoveries and personalized glucose management for individuals living with diabetes. Aiming toward standardization of definitions, calculations, and methods of usage for CGM metrics contributes to the development of an open science framework ensuring these tools are accessible and actionable for participants who value these metrics.

## Supplementary material

10.2196/92455 Checklist 1 PRISMA Checklist

## Abbreviations

%CV: glucose variability
ADRR: average daily risk range
AGP: ambulatory glucose profile
AUC: area under the curve
CGM: continuous glucose monitoring
CGP: comprehensive glucose profile
COGI: continuous glucose monitoring index
CONGA: continuous overall net glycemic action
CV: coefficient of variation
DRH: Diabetes Research Hub
GCI: glucose color index
GMI: glucose management indicator
GMI−HbA_1c_: acute glycemic gap
GRADE: glycemic risk assessment diabetes equation
GRI: glycemic risk index
HAS: hypoglycemia score
HbA_1c_: hemoglobin A_1c_
HBGI: high blood glucose index
IGC: index of glycemic control
IntHyper: intensity of hyperglycemia
IntHypo: intensity of hypoglycemia
LBGI: low blood glucose index
LI: lability index
MAGE: mean average glucose excursions
MBG: mean blood glucose
MODD: mean of daily differences
TAR: time above range
TBR: time below range
TIR: time in range
uGMI: updated glucose management indicator
